# Aphasia

**Published:** 1887-06

**Authors:** J. L. Gray


### APHASIA.

A CLINICAL LECTURE BY PROFESSOR J. A. ROBISON, DELIVERED AT COOK COUNTY HOSPITAL.

[Reported by J. L. Gray, M. D.]

The cases to which I wish to call your attention are in some respects unique. The first is that of a man aged 25, a clerk by occupation, who was admitted to the hospital March 11th, 1887. At the time of admission he was unconscious. There was paralysis of the right arm, the right lower extremity and *right*-sided facial paralysis. The pupils did not respond to light. In the course of 48 hours his consciousness returned, and while he could understand all questions that were asked, he was unable to speak, and of course unable to write, on account of the paralysis of the right arm. In a few days, however, he was able to write, using his left hand. All symptoms of paralysis in the lower extremity disappeared in 48 hours after admission. The facial paralysis disappeared in five days, but the paralysis of the right arm continues to the present time.

Three days after admission he was able to say "yes" and "no," His power of speech has gradually improved until at the present time he can speak with sufficient distinctness to make himself understood, after several attempts. For example, when he says "very good" the consonant "v" is "b," and "good" is "gut," sounding like "bery gut." When he says "thin" it is "hin." There has been at no time since his return to consciousness, any amnesic aphasia; it has been purely ataxic, this inability to speak certain words, although comprehending them perfectly. This patient previously had excellent health, although he has been a hard drinker, and his present disorders were probably caused by a protracted spree, but he has no recollection of how it commenced. He was found on the street, taken to the station and then brought to the hospital. In my own mind I have localized the lesion in the left frontal convolution. Although the patient is a young man, in all probability he had a small cerebral hemorrhage and at the same time areas of active congestion, giving rise to temporary paralysis.

The treatment has been complete rest in bed, good nourishment and no medicine whatever until yesterday, when he commenced taking 8 grains of potassium iodide and 15 minims of ergot three times daily.

The next patient is H. W., aged 25, a commercial traveler, and a very fine penman. Eleven months ago he had a chancre which was followed by the regular specific symptoms of sore throat, fever, eruption, etc., and he was placed on the mercurial treatment for syphilis.

March 1st, while attending to his business as a commercial traveler, he had "a stroke of paralysis" in which there was paralysis of the right side of the face, right arm and complete loss of power of speech. This was at first attended with unconsciousness, which, however, speedily passed away, and in the course of ten days the paralysis of the right arm also disappeared. At present there is slight paresis of the right facial muscles. His memory and mental faculties are unimpaired with the exception that he can not read as intelligently as before. When reading there are certain words which de does not seem to comprehend, and the type seems indistinct and blurred.

After the symptoms of paralysis of the right arm had disappeared he attempted to write, and although he was a very fine penman before the attack, it is with difficulty he can write his name, now. When he is requested to write any other words except his name he is unable to do so. He can write anything on the black-board, slowly, but cannot use a pencil.

The only words which he is able to articulate are "yes" and "no." In trying to speak he is very earnest, but in the endeavor to make himself understood, repeats frequently "I say," the sound being "I shay." He comprehends very distinctly everything that is said to him. The vowel A he can not articulate without joining it with a consonant. O and I he can articulate. The other vowels he can not. S, N and Sh are the only consonants he can articulate.

His treatment has been inunctions of the oleate of mercury until symptoms of ptyalism were produced, and internally the iodide of potassium. He seems to be in perfect health with the exception that he is unable to talk. He now has as much power in the right hand as he ever had. I shall try the effect of the same principles of education with this case as are employed with children, thus endeavoring to again place him where he will be able to write and talk.

I wish now to call attention to the case of a man about 27 years of age, who was brought into the hospital unconscious. There was spastic paralysis of the right arm and right lower extremity. There were also greatly exaggerated tendon reflexes, especially of the right side. His consciousness gradually returned in a few hours. When asked his name he was unable to give it, but wrote it on a piece of paper. We were unable to get any history whatever. In a few days he commenced to speak, and with the exception of dropping out a few words, would articulate a complete sentence rather distinctly. The trouble with his right arm and right lower extremity gradually disappeared, and in a week he could give a complete history of his life, and among other things, a clear history of his having had syphilis was obtained. In ten days from the time of admission he left the hospital apparently cured.

In eonclusion, gentlemen, I wish to remind you that you will not often see, either in hospital or private practice, three cases of ataxic aphasia all at about the same time, and all due to cerebral hemorrhage, syphilis being an important factor in two of the cases.

